# An evaluation of a multi-partner approach to increase routine immunization coverage in six northern Nigerian States

**DOI:** 10.1186/s12913-024-11403-3

**Published:** 2024-08-20

**Authors:** Leanne Dougherty, Mayokun Adediran, Akinwumi Akinola, Matthew Alabi, Eno-Obong Etim, Jane Ohioghame, Adebola Adedimeji

**Affiliations:** 1 Population Council, Abuja, Nigeria; 2 4301 Connecticut Avenue, NW, Suite 280, Washington, DC 20008 USA

**Keywords:** Partnership, Nigeria, Immunization, Health system strengthening, Accountability

## Abstract

**Background:**

Global health partnerships are increasingly being used to improve coordination, strengthen health systems, and incentivize government commitment for public health programs. From 2012 to 2022, the Bill & Melinda Gates Foundation (BMGF) and Aliko Dangote Foundation (ADF) forged Memorandum of Understanding (MoU) partnership agreements with six northern state governments to strengthen routine immunization (RI) systems and sustainably increase immunization coverage. This mixed methods evaluation describes the RI MoUs contribution to improving program performance, strengthening capacity and government financial commitment as well as towards increasing immunization coverage.

**Methods:**

Drawing from stakeholder interviews and a desk review, we describe the MoU inputs and processes and adherence to design. We assess the extent to which the program achieved its objectives as well as the benefits and challenges by drawing from a health facility assessment, client exit interview and qualitative interviews with service providers, community leaders and program participants. Finally, we assess the overall impact of the MoU by evaluating trends in immunization coverage rates.

**Results:**

We found the RI MoUs across the six states to be mostly successful in strengthening health systems, improving accountability and coordination, and increasing the utilization of services and financing for RI. Across all six states, pentavalent 3 vaccine coverage increased from 2011 to 2021 and in some states, the gains were substantial. For example, in Yobe, vaccination coverage increased from 10% in 2011 to nearly 60% in 2021. However, in Sokoto, the change was minimal increasing from only 4% in 2011 to nearly 8% in 2021. However, evaluation findings indicate that issues pertaining to human resources for health, insecurity that inhibits supportive supervision and vaccine logistics as well as harmful socio-cultural norms remain a persistent challenge in the states. There is also a need for a rigorous monitoring and evaluation plan with well-defined measures collected prior to and throughout implementation.

**Conclusion:**

Introducing a multi-partner approach grounded in a MoU agreement provides a promising approach to addressing health system challenges that confront RI programs.

## Introduction

Routine Immunization (RI) is the backbone of all national immunization programs and disease control efforts. The Government of Nigeria has faced persistent challenges in addressing low immunization coverage rates in the northern region. According to the 2015 National Nutrition and Health Surveys, children receiving the pentavalent vaccine in six northern states ranged from 4% in Sokoto to 33% in Kaduna [1]. Between 2008 and 2018, Demographic and Health Surveys (DHS) reported that the country’s RI coverage had stagnated nationally and deteriorated in the northern regions. The 2018 DHS estimated full immunization coverage to be only 31% nationally, and 20–23% in the northwestern and northeastern regions respectively [2]. These low coverage rates have been driven by several challenges such as, shortage of vaccines and supplies, poor community engagement, weak human resource system and harmonization of stakeholder efforts, inadequate ownership; systemic bottlenecks including sub-optimal funding by many state governments; weak cold-chain and vaccine logistics systems; and ineffective supportive supervision [3–6]. In the northern states specifically, the RI program and polio eradication campaign have faced historical boycotts at the local level driven by rumors (e.g., vaccinations causes HIV or sterility in young Muslim girls) and amplified by the political context [7]. One significant determinant of the poor performance and underlying restraints included a need for political commitment and accountability that contributed to weak financial support [8]. While the government and several development partners have deployed significant resources to improve RI coverage in Nigeria, the results often fell short of expectations due to weak harmonization of stakeholder efforts [9].

Over the last twenty years, global health partnerships have emerged as an important resource for health system strengthening and addressing public health challenges [10–12]. These partnerships bring together two or more organizations toward a common goal and often engage in advocacy, provide financing, and support technical or capacity strengthening efforts [13]. Previous studies have identified a number of benefits from these partnerships including increased coordination, reduced duplication of investments and activities, knowledge sharing and increased funding due to the establishment of a common platform that gains legitimacy and support [14, 15]. Despite these benefits, a number of criticisms have also been made about global health partnerships, including that they impose external priorities through the introduction of vertical disease programs that distract countries from focusing on health system strengthening, limit stakeholders’ voices in decision making, provide insufficient resources, and promote poor governance practices [16–18].

### Intervention description

In response to the challenges with the routine immunization program in northern Nigeria, the Bill & Melinda Gates Foundation (BMGF) and Aliko Dangote Foundation (ADF) forged a partnership with six northern state governments (Bauchi, Borno, Kaduna, Kano, Sokoto and Yobe) in a multi-year Memorandum of Understanding (MoU) partnership that aimed to strengthen RI systems and sustainably increase its immunization coverage. Unlike previous global health partnerships that operated in multiple countries or at a national level, these RI MoUs created six state-level public-private platforms on which to establish sustainable financing for RI, improve partner coordination and accountability, strengthen RI systems and, ultimately, increase vaccination coverage. Ultimately, the MoU aimed to increase vaccination coverage for DPT3 to 80% by the end of the agreement which, is the immunization coverage rate needed to achieve herd immunity to prevent the spread of the poliovirus. The first partnership in collaboration with the Kano state government was introduced in 2012 and later expanded between 2014 and 2016, as the governments of Bauchi, Borno, Yobe, Kaduna, and Sokoto states negotiated and signed RI MoUs with the foundations. The United States Agency for International Development (USAID) joined as a technical partner in Bauchi and Sokoto states. An important component of the RI MoU was the establishment of state managed RI program bank accounts (referred to as basket funds) whereby partners could contribute to the program. BMGF also provided technical assistance through its local partners, Solina Health, Chigari Foundation, and others. Key resources and sample documents for the MoU approach including a sample MoU, workplan and costing model are publicly available [19].

### RI MoU logic model

**Fig. 1:**
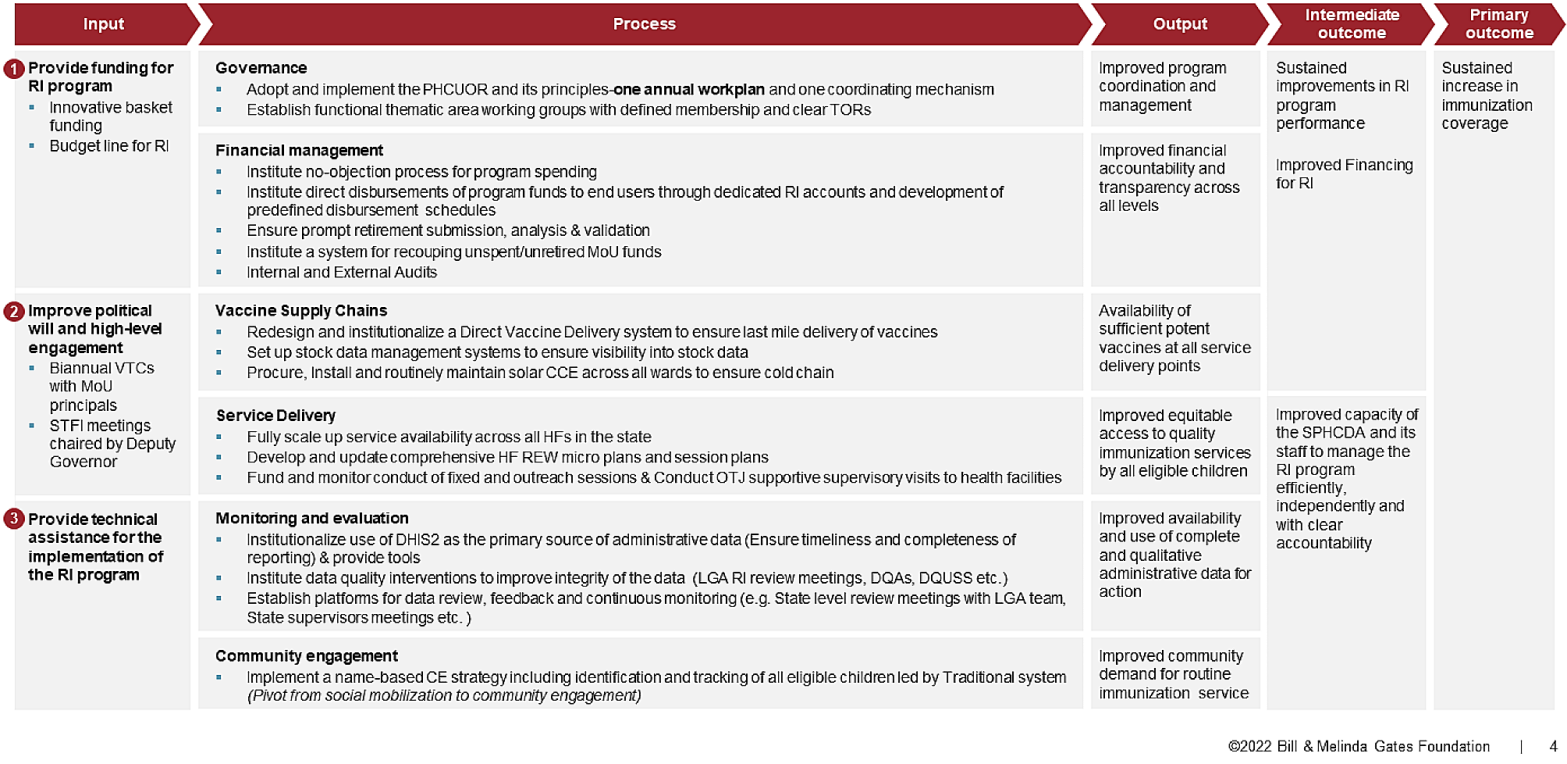
The RI MoU logic model

The MoU approach was developed with the primary outcome of increasing immunization coverage in six northern states of Nigeria by improving program performance and increasing capacity of the State Primary Health Care Development Agency (SPHCDA) and its staff to manage the program. To achieve these outcomes, inputs, as shown in Fig. 1, were focused on (a) the creation of a basket fund where resources from donors and government could be pooled together in a regressive funding model (i.e. BMGF and ADF contributed approximately 70% of RI program funds in the first year and then the proportion of funding declined each year as the state increased their contribution to fully fund the program by the end of the agreement), (b) establishment of meetings with key stakeholders and government officials to ensure high level government engagement, and (c) provision of technical assistance to support implementation of the program. The inputs aimed to facilitate processes related to governance, financial management, vaccine supply chain, service delivery, and monitoring and evaluation and community engagement that would ultimately improve (1) coordination and management, (2) financial accountability and transparency, (3) vaccine availability, (4) equitable access to quality services, (5) availability of quality administrative data for action, and (6) demand for RI services.

While there is some evidence on the benefits and challenges in implementing institutional health partnerships, there is limited evidence on their effectiveness [20]. In this paper, we contribute to the existing evidence base to evaluate the RI MoU partnership. We describe the extent to which the MoU was successful in achieving the desired objectives outlined in the RI MoU logic model and describe governments, donors and other stakeholders adherence to their financial commitments.

## Materials and methods

### Study setting

The MoU approach was implemented at the state, local government area (LGA) (i.e. an administrative subdivision of the state government) and health facility levels in six northern states. Population size across the six states ranges from approximately 13 million in Kano to three million in Yobe [21]. Several states (i.e., Borno, Kaduna and Sokoto) experienced insecurity during implementation.

### Study design

We conducted a mixed methods study to evaluate the effectiveness of the RI MoU approach in the six northern states. The rationale for the study approach was to (1) strengthen the level of inference for key findings by triangulating multiple data sources given the lack of baseline and comparison data values and; (2) provide a holistic understanding by capturing perspectives from multiple levels (e.g. participants, service providers, community structures, partners, donors and government). The evaluation was commissioned by the BMGF and conducted by the Population Council; an organization external to the MoU approach. First, to describe the MoU inputs and processes and adherence to design, the study team reviewed existing program documents through a desk review and conducted Key Informant (KI) interviews with stakeholders and state program implementers. Second, the study team conducted a quantitative health facility assessment and client exit interview and qualitative interviews with service providers, community leaders and program participants to assess the extent to which the approach achieved the objectives outlined in the RI MoU logic model and to understand the benefits and challenges of the approach. Finally, to assess the overall impact of the MoU in achieving program outcomes, we assessed data from household surveys and the District Health Information System 2 (DHIS2). The household survey data provided an objective assessment of RI program performance while the DHIS2 figures informed how well the system monitored and evaluated program performance. A summary of measures based on programmatic outputs and outcomes is described in Table 1.

**Table 1:** Summary of RI MoU evaluation outputs/outcomes

RI MoU Output	Measure/Domain	Data source(s)
Improved program coordination and management	• PHCUOR Implementation Scorecard average performance • Establishment of functional thematic working groups	• Desk review (i.e., PHCUOR scorecard 2015/2019) • KI interviews (2022)
Improved financial accountability and transparency across all levels	• Accountability measures • Percentage of health facilities reporting to receive funds at least quarterly • Percentage of health facilities completing an audit in the last 12 months	• Desk review • KI interviews (2022) • Health facility assessment (2022)
Availability of sufficient potent vaccines at all service delivery points	• Availability of functional refrigerators • Type of energy source for vaccine refrigerator • Primary supply chain mechanism for restocking supplies • Proportion all antigens stocked out or below minimum < 25% stock at apex health facilities	• Desk review (i.e., Vaccine dashboard (2021)) • KI interview (2022) • Health worker interviews (2022)
Improved equitable access to all immunization services by all eligible children	• Frequency of receiving supportive supervision • Percentage of caregivers who were told what vaccines were given to the child • Percentage of caregivers who were told the date for the next vaccination • Percentage of caregivers who reported provider wrote down the date for next vaccination appointment • Percentage of caregivers who were told about possible reaction or side effect	• Desk review (i.e., RISS dashboard) • Client exit interviews (2022)
Improved availability and use of complete administrative data for action	• Variance between survey and administrative coverage data pentavalent 3 • Health facilities with data collection systems in place	• Desk review (i.e., Analysis of DHS 2018 vs. DHIS2 2017& DHS 2021 vs. DHIS2 2020 • KI interviews (2022)
Improved community demand for routine immunization services	• Engagement with community actors • Percentage of caregivers who state the father is the primary decisionmaker related to child vaccination	• Client exit interviews (2022) • Health worker interviews (2022) • FGD community leaders (2022) • KI interviews (2022) • FGD program participants (2022)
Improved financing for RI	• Funds disbursed for the RI program by state government, BMGF and ADF by year	• Desk review (e.g., BMGF program documents)
Improved capacity of the SPHCDA and its staff to manage the RI program efficiently, independently and with clear accountability	• Median number of CHOs per health facility • Median number of midwives per health facility • Health worker motivation	• Desk review • Health worker interviews • Health facility assessment (2022)
Sustained increase in immunization coverage	• Proportion of children 12–23 months who received the pentavalent 3 vaccine	• NICS/MICS data from 2011, 2016, and 2021

### Desk review

Documents included in the desk review addressed all stages of the MoU design, start-up, and implementation. To inform analysis on the design of the MoU, the team reviewed the diagnostic reports, MoU agreements and case studies. To assess fidelity to implementation design, the team reviewed MoU related meeting summaries and presentations, workplans and strategies such as the community engagement strategy. To assess the extent to which the MoU achieved its objectives, the team reviewed findings from the national Primary Healthcare Under One Roof (PHCUOR) Implementation Scorecard, state financial management trackers, vaccine dashboards, routine immunization supportive supervision (RISS) monitoring, DHIS2 data to review fixed and outreach session completion, and AFeNET variance assessments between survey and administrative data. And, to assess impact of the MoU approach, the team reviewed National Indicator Cluster Survey/ Multiple indicator cluster survey (NICS/MICS) data from 2011, 2016, and 2021 to assess the proportion of children 12–23 months who received the pentavalent 3 vaccine.

### Quantitative data

**Fig. 2:**
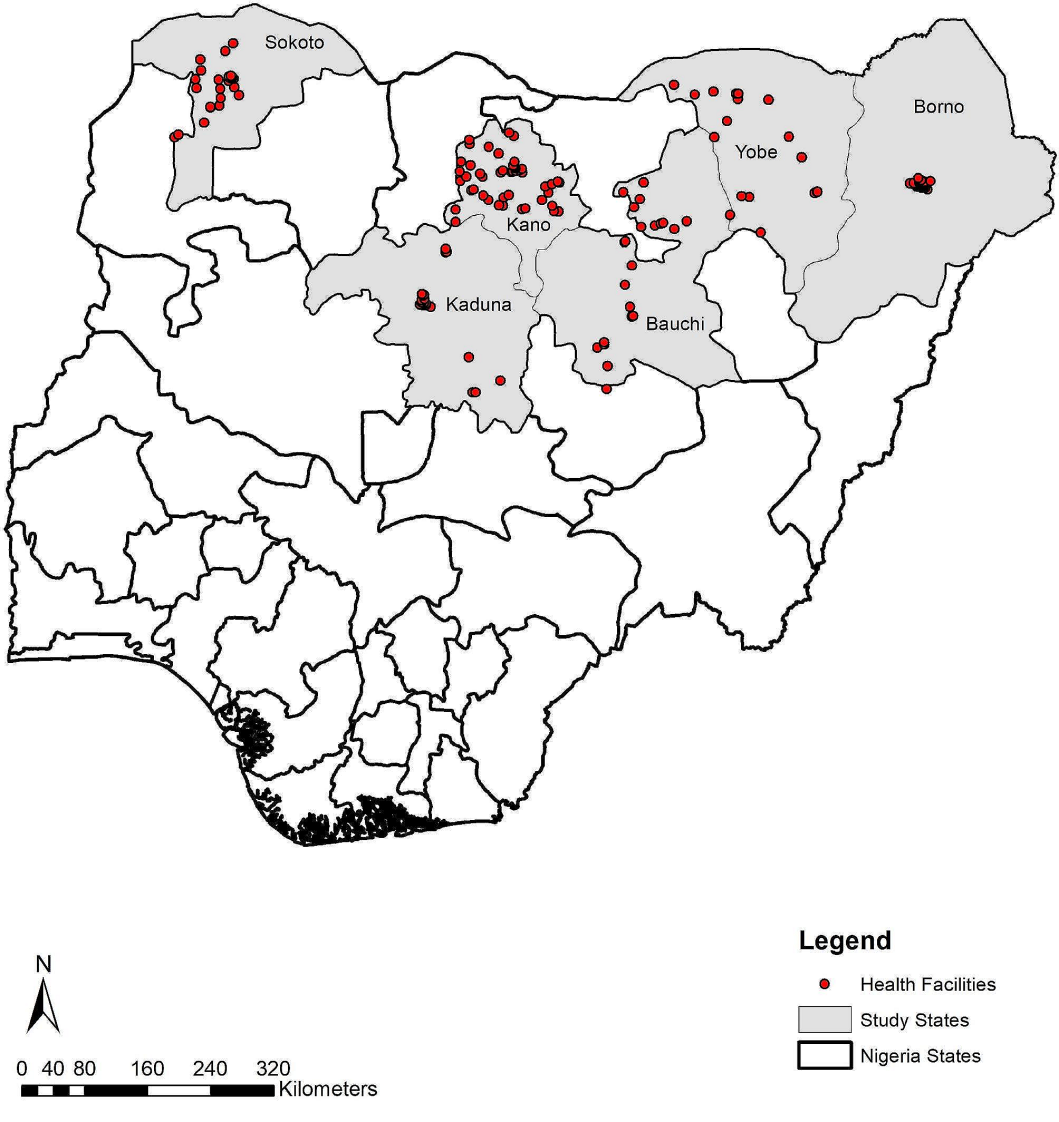
Map of Nigeria and study sites

A health facility assessment of Primary Health Centers (PHC) was conducted in March-April 2022 in selected local LGAs of the six implementation states (Supplementary file 1). We used a two-stage stratified sampling procedure in selecting health facilities. We generated a list of all the LGAs in each of the three senatorial districts and selected 50% of the total number of LGAs in each state with the exception of Borno and Kaduna due to security concerns as described in Table 2 and shown in Fig. 2. In Borno, the study team selected five LGA’s and oversampled health facilities within a secured area around the capital of Borno instead of the 14 LGAs as planned. In Kaduna, four LGAs near the capital of Kaduna were over sampled to replace LGAs in the western part of the state which could not be accessed due to security concerns. In each of the LGAs, approximately two PHCs were randomly selected. The targeted number of health facilities across all the LGAs was 156, at two health facilities per LGA. A total of 163 Health Facility Assessments (HFA) were conducted across all six MoU states. The additional seven health facilities were because of oversampling in Borno state due to the inability to access some LGAs because of security reasons. Client Exit Interviews (CEI) (*N* = 1,093) were conducted in the sampled health facilities to assess client satisfaction with RI services provided (Supplementary file 2). The study team interviewed on average 3–7 clients per facility except for in Yobe where immunization days were taking place resulting in a higher volume of clients referred for interviews. Clients were selected to participate in exit interviews if they were a primary caregiver (at least 18 years or older) of a child under the age of two years, accessing vaccination service.

**Table 2:** Sample size of health facilities and client exit interviews per state

States	Number of LGAs	Number of LGAs (planned)	Number of LGAs (actual)	Number of facilities	Number of client exit interviews
Bauchi	20	10	10	20	119
Borno	27	14	5	35	133
Kaduna	23	12	8	24	142
Kano	44	22	22	44	145
Sokoto	23	12	12	24	204
Yobe	17	8	8	16	348
**Total**	154	78	65	163	1,091

### Qualitative data

The qualitative data was collected in three separate data gathering activities between March and June, 2022. We conducted KI interviews with BMGF staff, government stakeholders, and implementers on the program model, execution, impact, and opportunities to be leveraged for future programs (Supplementary file 6). Second, In-depth Interviews (IDIs) with health workers were conducted to assess the extent to which the MoU achieved its objectives including benefits and challenges and lessons learned from the health worker perspective (Supplementary file 5). Lastly, Focus Group Discussions (FGDs) were conducted with community leaders (e.g. traditional, religious leaders) and program participants to assess benefits and challenges of the MoU and lessons learned from the community and program participants’ perspective (Supplementary files 3 and 4). KIs lasted approximately two hours while IDIs lasted approximately one hour in duration, both were conducted in English. FGDs lasted approximately 1–1.5 h in duration and were conducted in Hausa. Table 3 provides a summary of the interviews conducted by qualitative method and study respondent.

**Table 3:** Description of qualitative sample and method of selection

	Key Informant Interview	In depth interviews with health workers	Focus group discussions with community leaders	Focus group discussions with program participants
Description of study sample	Approximately 15 per state for a total of 91 interviews at the state level. State level participants included the State Immunization Officer, members of MoU Technical Working Groups, and implementing partners such as Chigari, Solina and USAID projects. An additional 10 interviews were conducted at the national/international level with BMGF Nigeria staff, BMGF US based staff, Solina, and several representatives of international organizations.	7 health workers were interviewed per state for a total of 42 respondents	Two FGDs per state for a total of 12 FGDs of 8 community leader participants	Two FGDs per state for a total of 12 FGDs of 8 mothers/caregivers of children under 2 years of age. One with caregivers adhering to the vaccination schedule and one with caregivers who have defaulted
Method of selection	Identified based on a listing of technical working group leadership or sub-leadership	Sampled one provider per facility selected for the quantitative survey	RI health workers identified community leaders and volunteers from community health facility committees	Participants were selected from health facility registers

### Analysis

For the quantitative data sources, we computed counts with percentages for categorical variables and medians with standard deviations for continuous variables. Data cleaning and analysis were performed using Stata version 14 software. For all of the qualitative data sources, the study team determined deductive codes prior to analysis based on the RI MoU logic model which draws from the World Health Organization health systems building blocks framework [22] and then generated subcodes inductively by reviewing transcripts line by line. Inconsistencies and questions that arose during coding were discussed through reoccurring meetings and resolved by consensus as a team to ensure inter-rater reliability. Sub-codes were further grouped during analysis to address research questions which included lessons learned, areas of improvement, recommendations, what worked well and what did not work well and sustainability. Additional codes were generated for the KIs conducted with national and international respondents and covered areas related to design, implementation, and transition. Codes for all of the qualitative data were analyzed thematically by state.

Study team members were responsible for managing specific data sources from data collection through analysis and used a convergence model of triangulation to bring together the complementary data sources during an analysis workshop conducted in June 2022. Study authors attended the analysis workshop. The objective of the workshop was to provide team members leading specific aspects of the study (e.g., qualitative interviews, desk reviews and quantitative surveys) an opportunity to present their respective findings by evaluation question and to discuss how each data source responded to the study questions and contributed to the overall evaluation findings.

The workshop was followed by regularly scheduled meetings where the team came together to discuss how each source responded to the study questions. We compared findings on similar topics and identified where different data sources worked to explain pathways outlined in the RI MoU logic model [23].

### Ethical approval

The study received approval from the Population Council Institutional Review Board (Protocol number 992). In Nigeria, ethical approval to conduct the study was obtained at national and state levels. At the national level, approval was granted by the National Health Research Ethics Committee with approval number NHREC/01/01/2007-17/01/2022. At the state level, ethics applications were submitted, and approval obtained from individual State Health and Research Ethics Committee before the commencement of field activity. Bauchi (NREC/03/11/19B/2021/10); Borno (073/2021); Kaduna (MOH/ADM/744/VOL.1/1171); Kano (SHREC/2022/3078); Sokoto (SKHREC/016/2022) and Yobe (MOH/GEN/747). The relevant ethical approval and consent details were received and are available on request by the editor or editorial office. Study participants provided informed consent by using their signature. In addition, all methods were carried out in accordance with relevant guidelines and regulations and with the 1964 Helsinki declaration and its later amendments or comparable ethical standards.

## Results

We present findings for each output considered in the RI MoU logic model specifically related to governance, financial management, vaccine supply chain, service delivery, and monitoring and evaluation and community engagement. Next, we consider outcome measures including the extent to which each partner met their financial contribution to the RI MoU and an assessment of immunization coverage rates over the period of implementation. A summary of the RI MoU performance measures by state is presented in Table 4.

**Table 4:** Summary of RI MoU Performance measures by State

Indicator	Source (date)	Bauchi	Borno	Kaduna	Kano	Sokoto	Yobe
1. *Governance*							
PHCUOR Implementation Scorecard average performance	PHCUOR Scorecard (2015)	67%	38%	46%	57%	45%	66%
PHCUOR Implementation Scorecard average performance	PHCUOR Scorecard (2019)	86%	60%	92%	76%	96%	97%
2. *Financial management*							
Percentage of health facilities reporting to receive funds at least quarterly	Health facility assessment (2022)	90%	80%	67%	89%	92%	88%
Percentage of health facilities completing an audit in the last 12 months	Health facility assessment (2022)	90%	60%	75%	75%	50%	94%
3. *Vaccine supply chain*							
Proportion all antigens stocked out or below minimum < 25% stock at apex health facilities	Vaccine dashboard (2021)	37%	92%	38%	41%	32%	41%
4. *Service delivery*							
Percentage of caregivers who were told what vaccines were given to the child	Client exit interviews (2022)	96%	59%	92%	90%	91%	85%
Percentage of caregivers who were told the date for the next vaccination	Client exit interviews (2022)	99%	89%	96%	94%	96%	85%
Percentage of caregivers who reported provider wrote down the date for next vaccination appointment	Client exit interviews (2022)	95%	51%	86%	59%	86%	62%
Percentage of caregivers who were told about possible reaction or side effect	Client exit interviews (2022)	98%	69%	84%	75%	92%	56%
5. *Community engagement*							
Percentage of caregivers who state the father is the primary decisionmaker related to child vaccination	Client exit interviews (2022)	48%	44%	25%	56%	27%	28%
6. *Monitoring and evaluation*							
Variance between survey and administrative coverage data pentavalent 3	DHS 2018 vs. DHIS2 2017	67%	45%	72%	51%	92%	74%
	NIC/MICS 2021 vs. DHSI2 2020	59%	63%	38%	51%	86%	29%
*7. Staff capacity*							
Median number of CHOs per health facility	Health facility assessment (2022)	2.0	1.0	2.0	1.0	2.0	1.0
Median number of midwives per health facility	Health facility assessment (2022)	0.0	1.0	1.0	1.0	1.0	1.0

### Governance: improved program coordination and management

The PHCUOR policy, enacted in June 2014 and considered a precondition for the MoU, called for states to consolidate planning and management around all PHC services and resources “under one roof,” the SPHCDA. The RI MoU aimed to support improved program coordination and management by encouraging adoption and implementation of the PHCUOR and its principles including the establishment of one annual workplan supported by the basket fund and one coordinating mechanism. The PHCUOR scorecard provides evidence of the state’s average performance across the nine PHCUOR pillars as shown in Table 4 under Governance. The nine pillars assessed governance and ownership, legislation, adoption of a minimum service package, repositioning, system development, operational guidelines, human resources, funding sources and structure and office setup. All six state governments improved their overall performance from 2015 to 2019 with a percentage point increase ranging from 19 to 51%.

The RI MoU also supported the establishment of functional thematic area working groups led by government employees with defined membership and clear terms of reference. The working groups addressed specific areas that needed strengthening, including finance, community engagement/social mobilization, monitoring and evaluation/supportive supervision, logistics, and training. This structure strengthened government ownership of the program contributing to strong political will for RI funding and creating partnerships to support program delivery.

*“Although it took time for everyone to come together and put it into one single workplan*,* but it has helped us to be operating as a single team. The spirit of teamwork was strengthened and visibility. So*,* even a new partner that comes*,* will have to now look at it and see where does your work key into this… So*,* [there is] integration in Yobe and [a] single work plan.” Government stakeholder*,* KI*,* Yobe*.

However, several challenges contributed to some delays in implementation including competing priorities between the national and state government as well as state government and partners.

*“The challenge sometimes is the approach from the partners. Sometimes what we want to do or intend to do is different from what the partners want us to do. although it is our own decision. We talked about this ownership*,* but sometimes this doesn’t exist when you look at it deeply. This is a challenge*,* really.” Government stakeholder*,* KI*,* Borno*.

In addition, political differences and interference in program implementation remains a challenge.

*“A politician … will just come and tell you*,* ‘Sir*,* appoint so person.’… If you didn’t appoint him then he will go back to the community and sabotage all your effort. And if you appoint him he may not be able to do the work you assign him to.” Government stakeholder*,* KI Sokoto*.

### Financial Management: improved financial accountability and transparency across all levels

The RI MoU instituted several processes to improve financial accountability and transparency across all levels of the health system. These processes included (1) instituting the no-objection process for program spending which stipulates that the state must seek approval from the MoU signatories to spend funds above a specified threshold; (2) instituting direct disbursements of program funds to end users through dedicated RI accounts and development of predefined disbursement schedules; (3) ensuring prompt retirement and submission, analysis and validation of funds; (4) instituting a system for recouping unspent/unretired MoU funds; and (5) conducting internal and external audits.

With the introduction of the MoU, bank accounts were opened at the state, LGA, and health facility level and the quarterly release and direct disbursement of funds was initiated. After the introduction of the MoU, the percentage of health facilities assessed which reported that funds were disbursed from state to health facility bank accounts at least quarterly ranged from 67% in Kaduna to 92% in Sokoto as shown in Table 4 under Financial management. Diagnostic reports conducted to inform the MoU design found that prior to the RI MoU funds disbursed were not regularly accounted for, and financial audits were not conducted on a regular basis. The MoU introduced financial management procedures including internal and external audits and aimed for all facilities to participate in a financial audit each year. As a result, the percentage of facilities assessed reported completing an audit in the 12 months preceding the assessment ranged from 50% in Sokoto to 94% in Yobe.

MoU partners considered the establishment of the basket fund to pool resources an effective mechanism that supported program coordination by eliminating multiple implementation silos and reducing duplication.

*“Having the funds in one basket has made it very nice… You have one channel of getting the resources*,* one channel again to implement all the programs…. unlike other ones when everybody is running his own parallel programs.” Government stakeholder*,* KI Kaduna*.

Through the introduction of the new MoU financial systems and processes, stakeholders also reflected on improved financial accountability and transparency.

*“We have introduced electronic digital financial tracking tool to ensure financial accountability*,* because you give people money to conduct an activity*,* if they don’t conduct the activity*,* they need to refund it. So*,* the executive secretary has made that every activity that has not been reported or that has been confirmed not to be conducted*,* the money must be refunded*,* and we have a lot of cases where people are refunding the money.” –Government stakeholder*,* KI*,* Yobe*.

However, some challenges still exist in the release of partners funds to support activities in the workplan due to conflicting fiscal years which has resulted in delays including quarterly disbursement from state to health facility bank accounts.

*“Although there are clear guidelines on how this funding should come but sometimes there is delay in the release of the funds. You have a beautiful plan*,* and it is time bound. You need to do this in January*,* you need to do 1*,* 2*,* 3*,* activities in the first week of February and the activities are costed and time bound. If the funds are not available as at when due*,* the activities may not take place.” Government stakeholder*,* KI Kano*.

### Vaccine supply Chain: availability of sufficient, potent vaccines at all service delivery points

The RI MoU implemented several measures aimed to redesign and institutionalize a Direct Vaccine Delivery system to ensure last mile delivery of vaccines. This included setting up stock data management systems to ensure visibility into stock data and procuring, installing, and routinely maintaining solar cold chain equipment (CCE) across all wards.

Prior to the MoU, there were routine stock outs and the diagnostic reports found that the method used to forecast vaccines which was based on target population and coverage was underestimating potential demand because LGAs were running out of stock by the end of the month. Following introduction of the MoU, the proportion of all antigens below the minimum level of adequate stock or stocked out at the apex health facilities ranged from 32% in Sokoto to 41% in Kano and Yobe in 2021 as shown in Table 4 under Vaccine supply chain. Several service providers noted that improved stock management further strengthened the state’s ability to make available sufficient, potent vaccines at all service delivery points.

*“I told you that we used to plan for how many vaccines we want*,* right? So*,* if … I didn’t plan for it*,* I didn’t know how many vaccines*,* how many doses of vaccines do I need… people will come waiting for me and at the end of the day I will say I didn’t have the vaccine or the vaccine has finished…There is one key form that we use to fill; that one you’ll fill it based on your vaccine consumption… They will not … just give me [vaccines] off head [without vaccine use data].” - Service provider*,* IDI*,* Kano*.

Adequate CCE was also procured through the MoU to ensure a more consistent supply of appropriately stored vaccines and fewer stockouts. The percentage of wards with functional cold chain equipment according to government managed vaccine dashboards increased across all six states. For example, in Bauchi, the percentage of wards with functional cold chain equipment was 96% in Bauchi in 2021 up from 28% in 2014–2015 at the time of MoU diagnostic assessments. Similarly, the percentage of wards with functional cold chain equipment was 93% in Sokoto in 2021 up from 29% in 2014–2015.

The increased availability of functional solar drives and other CCE strengthened the states’ ability to make available sufficient, potent vaccines at all service delivery points, as well as the use of third-party vendors for vaccine delivery in some states.

*“In every ward*,* we also have cold chain equipment. It’s also maintained by solar so there will be no wahala (problem) … We can keep [vaccines] at the local government level or at the facility level… if you go to anyone*,* they all have solar for maintenance of all our vaccines.” Government stakeholder*,* KI Bauchi*.

Initially, a push system for direct delivery of vaccines was introduced to apex health facilities through a private distributor to improve the reliability of vaccine delivery. With this approach, vaccines were distributed to bigger PHCs with Solar direct drive (apex facilities) in the community, from which smaller facilities or those without CCE – which rely on apex facilities for weekly vaccine supplies - may “pull” their supply. This eliminated high costs and long travel for personnel, as well as concerns about dangers associated with travel to collect vaccines. In Borno and Kaduna states, direct vaccine delivery through a third-party vendor alleviated pre-MoU challenges. However, in other states such as Bauchi, the government realized they had the capacity and means to effectively deliver vaccines and did not need to rely on a third-party distributor which was more expensive. In the end, facility readiness for CCE due to inadequate refurbishment by government, or theft, vandalism, or destruction of installed CCE, led most states to pursue a hybrid push-pull system for vaccine delivery despite initial focus on push system to apex facilities.

*“Previously*,* there were transportation issues around how we went to get the vaccines from the state’s cold chain officer… But presently it is easier because it is directly delivered by the vendors… making the routine immunization easier for us.” Service provider*,* IDI*,* Borno*.

However, insecurity, poor terrain, delays from the national level in vaccine delivery, and insufficient resources continued to pose problems in the timely delivery of vaccines to service delivery points.

*“During the rainy season*,* there are some areas you cannot go. We have hard to reach areas*,* no matter whatever the strategy you apply*,* you cannot get to that place. So*,* it’s a serious challenge.” Government stakeholder*,* KI Bauchi*.

### Service delivery: improved equitable access to quality immunization services for all eligible children

The RI MoU aimed to improve the equitable access to quality immunization services for all eligible children by fully scaling up service availability across all health facilities in the state; developing and updating comprehensive health facility reaching every ward (REW) microplans and session plans and funding and monitoring implementation of fixed and outreach sessions and supportive supervisory visits to health facilities. The investments contributed to high levels of planned fixed and outreach sessions conducted between 2017 and 2020 (data not shown). For example, planned supportive supervision visits that were conducted also increased in Kaduna from 55% in 2018 to 82% in 2020 as observed in the RI supportive supervision dashboard. Increases in planned supportive supervision visits conducted in Yobe also increased from 70% in 2018 to 84% in 2020.

The RI MOU also aimed to improve the quality of immunization services provided. Client exit interviews assessed the quality of provider-client interactions by asking if providers provided information on four important RI counseling points. In three states (Bauchi, Kaduna, and Sokoto), the percentage of clients who said providers shared information on the four RI counseling was over 85% as shown in Table 4 under service delivery. However, more variation was observed in Borno where the percentage of clients who said providers shared information on the four RI counseling was less than 70% for three of the four components.

### Monitoring and Evaluation: Improved availability and use of complete and quality administrative data for action

The RI MoU worked to institutionalize the use of DHIS2 as the primary source of administrative data by providing tools to ensure timeliness and completeness of reporting. Additional efforts included instituting data quality interventions to improve the integrity of the data and establishing platforms for data review, feedback, and continuous monitoring.

Evidence of improvements in data quality was identified in Bauchi where the variance between survey and administrative immunization coverage rates declined from 67% in 2017 for pentavalent 3 to 59% in 2020 as shown in Table 4 under Monitoring and evaluation. Similar trends were observed in other states such as Kaduna where the variance declined from 72% in 2017 for pentavalent 3 to 38% in 2020. The establishment of frequent data review meetings has contributed to the improvements in data quality as well as digitized supportive supervision improved monitoring efforts.

*“What worked well is that we’ve already digitalized our supervision because they are in the same group. Now we are using digital devices to do supervision*,* it’s faster*,* easier and it has the coordinates*,* unlike before where somebody will sit down on his bed and fill a form that he has gone to supervision. Now when you send our report it will show the coordinates where you sent [it from]. – Government stakeholder*,* KI Kano.*

Despite these improvements, challenges remain with data reporting including falsification of monitoring data as described by one government stakeholder, an overburdened workforce, and issues with security.

*“The challenges we are facing is that health workers are … overstretched with a lot of activities. And they tend to see the data reporting as not important as the rendering of the services. So…in reporting*,* they tend to be negligent in some of the activities.” Government stakeholder*,* KI Yobe.*

### Community Engagement: improved community demand for routine immunization services

The RI MoU aimed to improve community demand for RI services by implementing a name-based community engagement strategy including identification and tracking of all eligible children led by a traditional system. All states adopted the use of line listing for newborn, as well as defaulter tracking to improve community use of vaccination services. States worked with community actors (Mai Unguwas) and created defined roles and plans to support the work. This engagement with community actors was seen as an important contribution to the RI program.

*“We used to call the community stakeholders*,* telling them the importance of immunization*,* and we use to tell them the importance of their participation for these services. So that helps us a lot. They used to go for community mobilization. They are having meeting within themselves to mobilize their people that they should come and take this vaccine because the vaccine is very important.” Service provider*,* IDI Bauchi.*

Based on client exit interviews, mothers are not the primary decision maker regarding the child’s vaccination status in a number of states. For example, in Kano, 62% of fathers are the primary decision makers regarding whether the child goes for vaccinations as shown in Table 4 under community engagement. This challenge was reflected in the qualitative interviews, where a program participant noted that women cannot access services without the husband’s approval.

*“The woman cannot do anything about [service uptake] if her husband is against it.” Program participant*,* FGD Bauchi*.

However, there is some evidence that religious leaders are engaging with both men and women to encourage the use of vaccination services.

*“We both know we’re in the North. I mean not just even in the North*,* Nigeria as a whole*,* we tend to really listen to our religious and traditional leaders… Carrying along those institutions that we knew that could have some influence on the people was also something that went well.” Partner*,* KI Kaduna*.

While community leaders played an important role, the lack of incentives for community volunteers and influencers resulted in poor motivation to conduct activities.

*“They [volunteers] are not on a pay roll. And you know the economic situation in…not only the state*,* in the country… Some are still volunteering to do the job*,* but some are saying since there is no pay*,* we are not continuing.” Government stakeholder*,* KI Kaduna*.

This is further supported with qualitative evidence where spousal refusal from poor sensitization on adverse events following immunization, contributed to vaccine hesitancy and refusal.

*“The children get fever when we return. When he [husband] asked why and I told him that I collected an injection for them*,* he asked why? I should not go again; that on the quest of getting drugs for catarrh*,* I have brought something new [and more serious] upon him.” Non-beneficiary*,* FGD Borno*.

### Improved Financing for RI

The MoU approach was developed with the goal of creating sustained financing for the RI program. We assessed MoU funds contributed between 2013 and 2022 by contributor. Overall, BMGF and ADF paid the full amount of their committed funds over the course of the MoU period for all six states. However, each state made varied progress towards their commitment of assuming the full program costs as shown in Table 5. Bauchi state paid $3.8 million of the $6.5 million U.S. Dollar (USD) committed from 2015 to 2018 while Kaduna state paid $3.5 million of the $4.6 million USD committed from 2016 to 2021. Kano paid $7.8 million of the $12.5 million USD committed from 2013 to 2021 while Yobe paid $2.8 million of the $4 million USD committed from 2016 to 2021. Borno paid $1.8 million of the $2.6 million USD committed while Sokoto paid $2.6 million of the $3.8 million USD committed both from 2016 to 2021.

**Table 5:** Percentage of RI MoU annual funds contributed by State government, BMGF and ADF, by year

		**2015**	**2016**	**2017**	**2018**	**2019**	**2020**
Bauchi	State	30	50	71	76	100	100
	ADF	35	25	15	12		
	BMGF	35	25	14	12		
		**2016**	**2017**	**2018**	**2019**	**2020**	**2021**
Borno	State	9	18	40	53	70	70
	ADF	45	41	30	11	15	15
	BMGF	45	41	30	36	15	15
		**2016**	**2017**	**2018**	**2019**	**2020**	**2021**
Kaduna	State	30	50	70	70	70	82
	ADF	35	25	15	15	15	18
	BMGF	35	25	15	15	15	0
		**2013**	**2014**	**2015**	**2016**	**2017**	**2018–2021**
Kano	State	32	41	83	66	100	100
	ADF	34	29	8	17		
	BMGF	34	29	8	17		
		**2016**	**2017**	**2018**	**2019**	**2020**	**2021**
Sokoto	State	30	50	74	70	70	70
	ADF	35	25	13	15	15	15
	BMGF	35	25	13	15	15	15
		**2016**	**2017**	**2018**	**2019**	**2020**	**2021**
Yobe	State	30	42	78	78	78	69
	ADF	35	22	11	11	11	15
	BMGF	35	36	11	11	11	16

### Improved capacity of the SPHCDA and its staff to manage the RI program efficiently and independently and with clear accountability

The MoU aimed to improve the capacity of the SPHCDA and its staff to manage the RI program. Management capacity to implement the MoU was built through trainings and learning visits to Kano. Cascade trainings were implemented and built capacity to deliver RI services, manage cold chain, improve monitoring and evaluation, etc. and, the Basic Guide for RI Service Providers was introduced and used to train staff and serve as a reference document.

The HFA found that in most states, the National Primary Health Care Development Agency (NPHCDA) minimum standard number of at least two community health officers (CHO) and five community health extension workers (CHEWs) were not available. For example, in Bauchi, there was a median of two CHOs at the facility and there were no full-time nurses or midwife assigned to the facility as shown in Table 4 under Staff capacity. Community health worker motivation was another challenge reflected through the qualitative interviews.

*“The challenge is that they need some incentives*,* then some support either financially or what have you*,* most especially those volunteers. They are not having salaries; their work is voluntary. So sometimes*,* they may demand some assistance*,* and we use to take it into consideration*,* but the authority concerned cannot do it.” Service provider*,* IDI Bauchi*.

However, efforts to improve health worker performance through appraisals, rewards, and annual recognitions was seen as a promising approach.

Outside urban areas, insufficient numbers of skilled health workers to meet rising demand for services, and dwindling state resources to hire health workers and pay their salaries was mentioned as a challenge.

*“There [are] dwindling resources to employ. Even when you want to employ the satisfactory number of health workers into the facilities…sometimes their salaries and wages is something …we’ve been having these challenges of resources.” Government stakeholder*,* KI Kaduna*.

### Sustained increase in immunization coverage

**Fig. 3:**
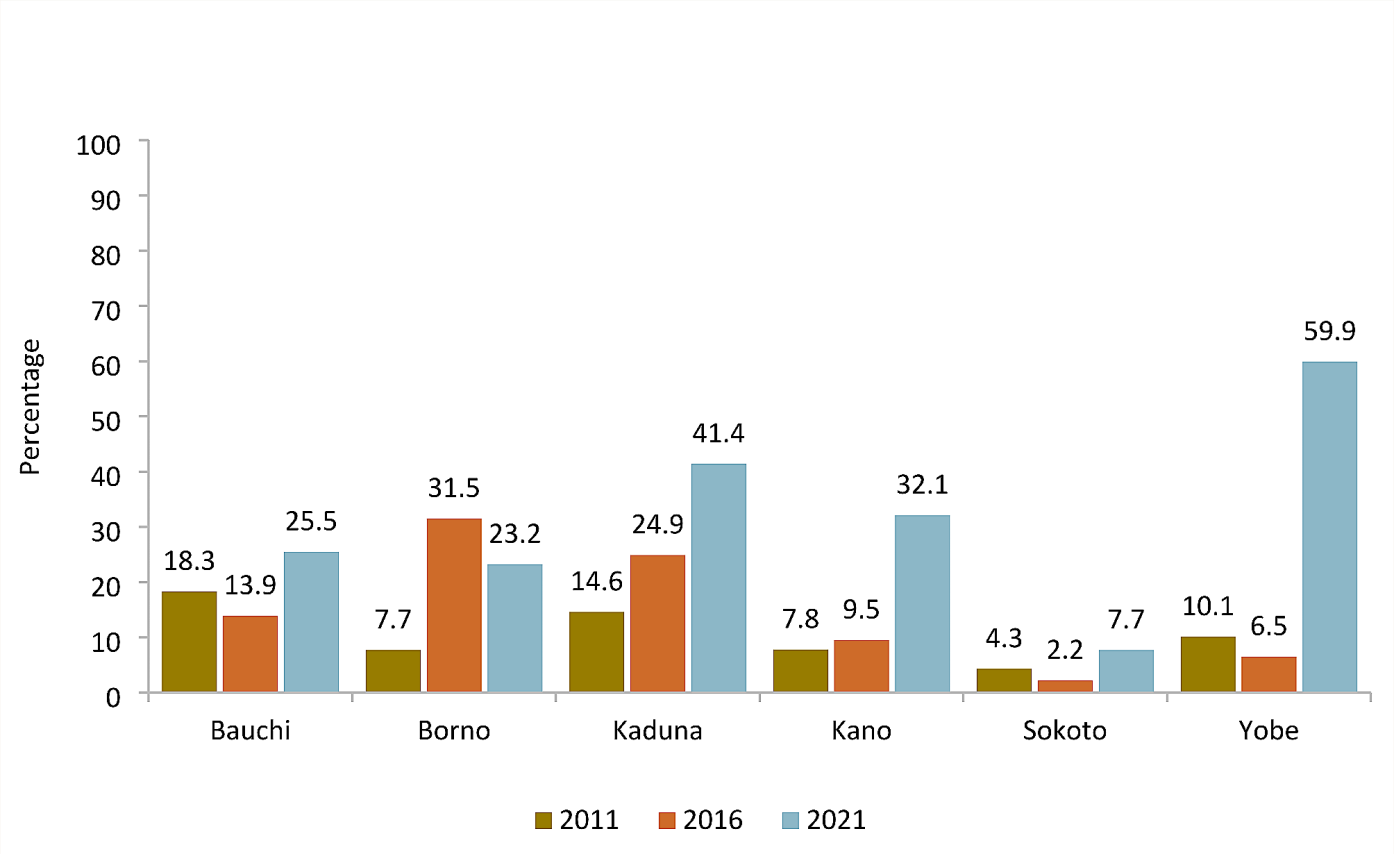
Proportion of children 12–23 months who fully vaccinated by MoU State, 2011, 2016, 2021

The MoU approach was developed with the primary outcome of increasing immunization coverage. Figure 3 presents the proportion of children 12–23 months who were fully vaccinated. Across all six states, vaccination coverage increased from 2011 to 2021. In some states, the gains were substantial. For example, in Yobe, vaccination coverage increased from 10% in 2011 to nearly 60% in 2021. However, in Sokoto, the change was minimal increasing from only 4% in 2011 to nearly 8% in 2021.

## Discussion

This evaluation explains the extent to which the RI MoU contributed to improved program performance including increased immunization coverage through strengthened health system capacity and increased government financial commitment across six states in northern Nigeria. Our findings, organized by RI MoU logic model, contribute to the growing body of literature exploring how global health partnerships can be used to strengthen public health programs. Drawing from multiple data sources over the course of implementation, we assessed measures of program performance and identified benefits and challenges associated with implementation.

Several notable achievements associated with RI MoU investments were observed. First, we found the RI MoU was successful in instituting mechanisms that improved coordination across partners and increased government ownership of decisions which was consistent with previous research [24]. There was also evidence from the PHCUOR scorecard assessment of progress in addressing the PHCUOR pillars. However, state governments addressed specific pillars to varying degrees which may be consistent with previous findings that state governments are more interested in executing aspects of the PHCUOR that are easy to achieve rather than address the more challenging human resource management and funding requirements [25, 26]. The RI MoU was also successful in improving financial accountability and transparency. Previous research has found a lack of accountability and widespread corruption to be a barrier to high RI program performance in Nigeria [27, 28]. However, evaluation findings found evidence that investments led to improvements in accountability and transparency because of the introduction of electronic mechanisms for validating expenditures as well as establishing routine audits. The significant investment in upgrading the vaccine supply chain including the procurement of solar refrigerators and direct to facility deliveries of vaccine supplies has also contributed to a reduction in stock out rates [29]. However, insecurity remains a challenge in some areas resulting in the destruction of installed CCE in some wards and more effort is required to work directly with community leaders to protect installed CCE.

RI MoU states showed improvement in vaccination coverage rates from 2011. However, there was variation between states suggesting that challenges remain. First, the RI MoU community engagement efforts focused largely on line listing approaches to encourage uptake of vaccination which may not have been adequate to address the numerous challenges at the community level. The approach required strong support from community leaders and the use of community volunteers who were not compensated for their services. Introducing non-monetary incentives may be an effective option to increase community based participation and motivation for the RI program [30]. While the use of community leaders is important in addressing community level barriers [31], the approach did not focus on the individual behavioral barriers that may require efforts to address knowledge, attitudes, beliefs, social norms, and self-efficacy. Efforts such as SMS reminders may be one way to raise awareness about vaccination services [32]. In addition, efforts to engage directly with fathers who were often the primary decision maker of whether a child was vaccinated through traditional channels such as Wanzams may also address barriers to vaccination coverage [33, 34].

In addition to challenges at the community level, a number of challenges were noted relating to service delivery and staff capacity. Data from the HFA found that the median number of health workers was below the recommended number at PHCs in all states. Given the limitations of the public sector to provide services due to insufficient staff as well as challenges in reaching insecure areas, it may be beneficial to consider a public-private partnership model to expand service delivery [35] to hard-to-reach areas or consider redistributing and/or incentivizing staff to work in hard to reach areas. In addition, given the frequent migration and political insecurity, it may be necessary to adopt new methods such as applying satellite derived maps to identify vulnerable populations that are not being reached through traditional RI microplan approaches [36–38]. Finally, training health providers on how to address vaccine hesitancy and concerns related to adverse events following immunization may also be required [39].

Several efforts were made to improve routine monitoring of the RI program including the incorporation of RI module in DHIS2 which provided information to inform planning [40–42]. However, insecurity remained a challenge in some areas compromising monitoring and supportive supervision visits in some local governments, which has led to poor data reporting. Consequently, the state must explore innovative approaches to retrieve program data from high-risk security areas [43]. Finally, the RI MoU logic model provided a structure for assessing the RI MoU performance, however a theory driven model including a clear monitoring and evaluation plan with indicators and targets established prior to implementation of the MoU is required in order to provide a more rigorous assessment of the RI MoU approach [44, 45]. This approach would also help to provide a better understanding of why some states such as Yobe have been effective in increasing immunization coverages while others such as Borno and Sokoto continue to struggle.

### Limitations

There are several limitations associated with this study. First, each state included in the evaluation initiated implementation at different time points and progressed to more comprehensive PHC MoU models at staggered times. In addition, while the states completed a comprehensive diagnostic assessment prior to implementation, comparable baseline measures were not collected. Next, due to the complexity of the approach, no single data source could be used to measure the full influence of the approach. This coupled with the lack of a comparison area made it impossible to control for the effect of individual inputs or how the MoU states performed relative to other states in the region who did not benefit from the MoU investment [20]. Third, a comprehensive monitoring and evaluation plan was not established prior to implementation with clearly defined indicators and data sources. As such, limited data were available following several years of implementation and at irregular intervals. Finally, details on programs not operating under the purview of the RI MoU approach as well as contextual factors likely influenced the variable outcomes observed across the six states [46]. Specifically, Gavi’s national level investments to strengthen the health system including cold chain equipment investment despite focusing on non-MoU states may have reached to a limited extent MoU states. And, the Covid-19 pandemic while not assessed in this evaluation contributed to a lack of transport and limited outreach visits [47]. Despite these challenges, the evaluation did endeavor to achieve a holistic understanding of the RI MoU approach by gathering perspectives from multiple levels (e.g. participants, service providers, community structures, partners, donors and government stakeholders).

## Conclusion

Consistent with previous research on the advantages of partnership models, we found the RI MoUs across the six states to be mostly successful in strengthening health systems, improving accountability and coordination, and increasing the utilization of services and financing for RI which serves as an important foundation as the country transitions to sector wide approaches [14, 15]. However, evaluation findings indicate that issues pertaining to human resources for health, insecurity that inhibits supportive supervision and vaccine logistics as well as harmful socio-cultural norms remain a persistent challenge in the states suggest that the RI MoU approach would benefit from increased technical assistance and capacity building to address these limitations [13]. Attention to numbers, capacity, and distribution of frontline health providers will be an important component for health system strengthening moving forward. Furthermore, addressing cultural norms received minimal consideration throughout the MoU design. If interventions to address socio-cultural norms are not incorporated into the program, service uptake may remain low.

## Data Availability

The datasets generated and/or analysed during the current study are not publicly available to protect the confidentiality of study participants but are available from the corresponding author on reasonable request.

## Abbreviations

ADF: Aliko Dangote Foundation
BMGF: Bill and Melinda Gates Foundation
CCE: Cold Chain Equipment
CHEW: Community Health Extension Worker
CHO: Community Health Officer
CEI: Client Exit Interview
DHIS2: District Health Information System 2
DHS: Demographic and Health Survey
FGD: Focus Group Discussion
HFA: Health Facility Assessment
IDI: In depth Interview
KI: Key Informant
LGA: Local Government Area
MoU: Memorandum of Understanding
NPHCDA: National Primary Health Care Development Agency
PHC: Primary Health Care
PHCUOR: Primary Health Care Under One Roof
REW: Reaching every ward
RI: Routine immunization
RISS: Routine Immunization Supportive Supervision
SDD: Solar Direct Drive
SPHCDA: State Primary Health Care Development Agency
USAID: U.S Agency for International Development
USD: United States Dollar
